# Effects of substrate color on intraspecific body color variation in the toad‐headed lizard, *Phrynocephalus versicolor*

**DOI:** 10.1002/ece3.5545

**Published:** 2019-08-15

**Authors:** Haojie Tong, Jiasheng Li, Yubin Wo, Gang Shao, Wei Zhao, Diana Aguilar‐Gómez, Yuanting Jin

**Affiliations:** ^1^ College of Life Sciences China Jiliang University Hangzhou China; ^2^ School of Life Sciences Lanzhou University Lanzhou China; ^3^ Center for Computational Biology University of California Berkeley CA USA

**Keywords:** adaptation, digital photography, melanic, morphological color change, spectrometry, squamate

## Abstract

Diversity in animal coloration is generally associated with adaptation to their living habitats, ranging from territorial display and sexual selection to predation or predation avoidance, and thermoregulation. However, the mechanism underlying color variation in toad‐headed *Phrynocephalus* lizards remains poorly understood. In this study, we investigated the population color variation of *Phrynocephalus versicolor*. We found that lizards distributed in dark substrate have darker dorsal coloration (melanic lizards) than populations living in light substrates. This characteristic may improve their camouflage effectiveness. A reciprocal substrate translocation experiment was conducted to clarify the potential role of morphological adaptation and physiological plasticity of this variation. Spectrometry technology and digital photography were used to quantify the color variation of the above‐mentioned melanic and nonmelanic *P. versicolor* populations and their native substrate. Additionally, substrate color preference in both populations was investigated with choice experiments. Our results indicate that the melanic and nonmelanic populations with remarkable habitat color difference were significantly different on measured reflectance, luminance, and RGB values. Twenty‐four hours, 30 days, and 60 days of substrate translocation treatment had little effects on dorsal color change. We also found that melanic lizards choose to live in dark substrate, while nonmelanic lizards have no preference for substrate color. In conclusion, our results support that the dorsal coloration of *P. versicolor,* associated with substrate color, is likely a morphological adaptation rather than phenotypic plasticity.

## Graphical abstract

Melanic and nonmelanic populations with remarkable habitat color difference were significantly different on measured reflectance, luminance, and RGB values. Substrate translocation treatment had little effects on dorsal color change. Melanic lizards choose to live in dark substrate, while nonmelanic lizards have no preference for substrate color.

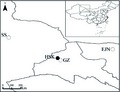

## 1 INTRODUCTION

Diversity of coloration is a widespread phenomenon in the animal kingdom. Color variation can help both predators and prey. It can benefit predators by making them less visible to the prey by camouflaging (crypsis). Similarly, it can help preys avoid predators by coloration crypsis or the opposite, by displaying bright conspicuous colors as a warning of unprofitability (aposematism; Cadena, Smith, Endler, & Stuart‐Fox, 2017; Cook, Grant, Saccheri, & Mallet, 2012; Edelaar, Baños‐Villalba, Escudero, & Rodriguez‐Bernal, 2017; Mallarino, Linden, Linnen, & Hoekstra, 2017; Stuart‐Fox & Moussalli, 2009). Coloration is also involved in courtship or signal transmission (Keren‐Rotem, Levy, Wolf, Bouskila, & Geffen, 2016; Ligon, 2014) and body temperature regulation (Jin, Tong, & Zhang, 2016; Langkilde & Boronow, 2012). Therefore, coloration plays a significant role for animals to adapt to variable environments.

Camouflage is a key for animals to survive in different color backgrounds. To display a feature of camouflage, it is of great importance for animals to select a substrate that blends with their body coloration. In addition to the fixed color adaptations (Hoekstra, Hirschmann, Bundey, Insel, & Crossland, 2006; Rosenblum, Roempler, Schoeneberg, & Hoekstra, 2010), selective pressure on behavioral preference of local color matching remains to be explored to understand the benefits of color adaptation. As the visual background against which it is viewed is the primary basis of camouflage, various species prefer substrate or habitat that enhance matching for camouflage, including fish (Kelley, Taylor, Hart, & Partridge, 2017; Kjernsmo & Merilaita, 2012; Smithers, Rooney, Wilson, & Stevens, 2018), amphibians (Polo‐Cavia, Miguel Oliveira, Redondo Villa, & Marquez, 2016; Rabbani, Zacharczenko, & Green, 2015), reptiles (Hamilton, Gaalema, & Sullivan, 2008; Marshall, Philpot, & Stevens, 2016; Nafus et al., 2015), birds (Lovell, Ruxton, Langridge, & Spencer, 2013), and insects (Gillis, 1982; Kang, Stevens, Moon, Lee, & Jablonski, 2015; Kettlewell & Conn, 1977; Sargent, 1966). Therefore, animal substrate color selections could be indicative of whether they are behaviorally segregated according to their body color and further explain its relationship with camouflage.

The skin of reptiles is generally considered to have three layers of pigment‐containing and light‐reflecting cells (chromatophores). The melanophore is in the third layer of skin, which is responsible for the synthesis of melanin (Rosenblum, Hoekstra, & Nachman, 2004). Two principal types of animal color changes have been reported: physiological and morphological color change. Physiological color change is caused by the movement (aggregation or dispersion) of pigment granules within chromatophores, often taking place in milliseconds to hours in response to short‐term environmental stimuli, showing a strong plasticity on animals’ body color (Nery & Castrucci, 1997; Sköld, Aspengren, Cheney, & Wallin, 2016; Sköld, Aspengren, & Wallin, 2013). In contrast, morphological color change occurs due to changes in the pigment production of chromatophores and takes up to several days or months in response to long‐term environmental stimuli. It is generally considered to be long‐term adaptation to a certain background (Kraemer, Kissner, & Adams, 2012; Rosenblum, 2005). Since the short‐term plasticity or long‐term adaptation would, respectively, result in physiological or morphological color change, the type of change within a specific organism needs to be clarified to point out the direction for future research.

Many reptiles will match their body coloration to habitat environments, including turtles (Hall, Robson, & Ariel, 2018; McGauch, 2008; Rowe, Bunce, & Clark, 2014; Rowe, Miller, et al., 2014; Xiao et al., 2016), snakes (Rajabizadeh, Adriaens, Kaboli, Sarafraz, & Ahmadi, 2015), gecko (Vroonen, Vervust, Fulgione, Maselli, & Damme, 2012), and lizards (Corl et al., 2018; Hamilton et al., 2008; Krohn & Rosenblum, 2016; Stuart‐Fox, Moussalli, & Whiting, 2008; Tao et al., 2018). These animals can adapt to varied substrate coloration in physiological and/or morphological color change. Hence, the implement of experiments on reciprocal translocation by changing the substrate coloration can be important to clarify the underlying role of physiological plasticity and morphological adaptation on body color variation.

**Figure 1:**
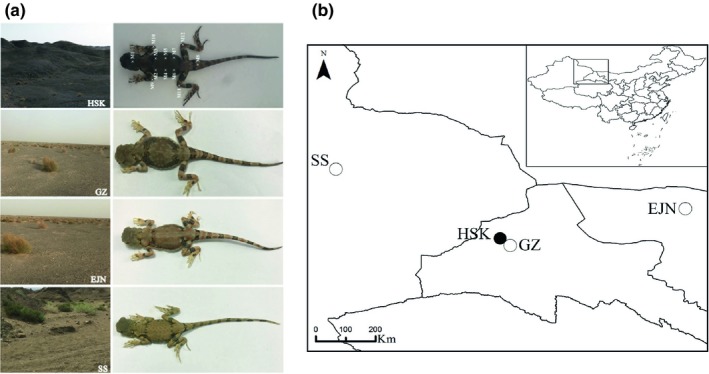
Sampling sites, body color, and substrates color of *Phrynocephalus versicolor* populations. (a) Black substrate and melanic lizards in HSK (Heishan Kou), weathered yellow substrate and nonmelanic lizards in GZ (Guazhou city), EJN (Ejin Banner), and SS (Shanshan city). M1–M12 marked on the melanic lizard represent twelve color measuring points for each lizard, including cranial center (M1), top left on the back (M2), top right on the back (M3), left side on the central back (M4), right side on the central back (M5), bottom left on the back (M6), bottom right on the back (M7), tail root (M8), left forelimb (M9), right forelimb (M10), left hind limb (M11), and right hind limb (M12). (b) Map shows the sampling sites of different *P. versicolor* populations

The *Phrynocephalus* lizards have received ecological attention on color variation in recent years. The central black abdomen existing only in high altitude populations of *P. theobaldi* was discovered by Jin and Liao (2015), and their subsequent study further confirms that this trait is associated with thermoregulation in cold regions (Jin et al., 2016). Sexual color dimorphism was reported in *P. guinanensis* (Ji, Wang, & Wang, 2009; Zhang, Tong, Wo, Liu, & Jin, 2018). Moreover, Tong et al. (2016) described the dorsal gray color variation between *P. versicolor* and *P. frontalis*. However, little is known about intraspecific color variation associated with habitat color changes in this genus. *Phrynocephalus versicolor* widely inhabits the deserts and semideserts endemic in eastern Xinjiang, western Inner Mongolia, western Gansu, and Ningxia province in China (Zhao, Zhao, & Zhou, 1999). We observed the dorsal coloration of *P. versicolor* distributed only at dark substrate in Liuyuan town (Gansu province) is visibly darker (melanic, see Figure 1a) than other conspecific populations (nonmelanic, see Figure 1a) living in weathered yellow (light) substrate. In this study, spectrometry technology (Matthews, Goulet, Delhey, & Chapple, 2016; Rowe, Bunce, & Clark, 2014; Rowe, Miller, et al., 2014) and digital photography (McGauch, 2008; McKay, 2013; Stevens, Parraga, Cuthill, Partridge, & Troscianko, 2007) were used to quantify the natural dorsal color variation and their native substrate color difference between melanic and nonmelanic *P. versicolor* populations, aiming to determine: (a) whether the color properties of melanic and nonmelanic lizards differed significantly; (b) whether the dorsal color properties from different habitats were associated with the different color substrates (i.e., black and weathered yellow sand), which could suggest background matching camouflage; and (c) whether each lizard color morph displays a behavioral preference for substrates that match their body coloration.

## 2 MATERIALS AND METHODS

### 2.1 Sampling

In May, 2017, our first sampling trip, a total of 45 (18 males, 27 females) melanic *P. versicolor* adults were collected from black mountainous area of Heishan Kou (HSK, 96.17°E, 41.08°N) in Liuyuan town, Gansu, China with an altitude of 1,235 m above sea level (m a.s.l.). Its substrate ground is mainly composed of exposed black stones, rich in iron elements. A total of 78 (22 males, 56 females) nonmelanic *P. versicolor* adults were respectively collected from Shanshan city, Xinjiang Uygur Autonomous Region (SS, 90.35°E, 43.24°N, 1,502 m a.s.l., 11 males, 33 females), and Ejin Banner, Inner Mongolia Autonomous Region (EJN, 100.88°E, 41.98°N, 933 m a.s.l., 11 males, 23 females). Weathered yellow gravel is the main substrate ingredient in these areas (Figure 1). In July, 2018, our second sampling trip, a total of 20 nonmelanic *P. versicolor* adults were collected from Guazhou county, Gansu, China (GZ, 95.61°E, 41.05°N, 1,386 m a.s.l., 11 males, 9 females) and 24 melanic *P. versicolor* adults (6 males, 18 females) from HSK. All adult lizards were collected within four days of fieldwork for each trip.

The melanic population was only observed in HSK, where the substrate is black. We found three nonmelanic populations had similar dorsal color (nonmelanic) and substrate color (light), despite the large varied elevation and geographical distance that separates them (Figure 1). All the collected individuals were transported and fed in the zoology laboratory of Lanzhou University within 24 hr after captured, and all lizards used in this work were individually marked (toe‐clipped) to give their ID. After the experiments were performed, they were safely released back into the wild at their captured places.

### 2.2 Feeding and reciprocal translocation experiments in artificial laboratory conditions

**Figure 2:**
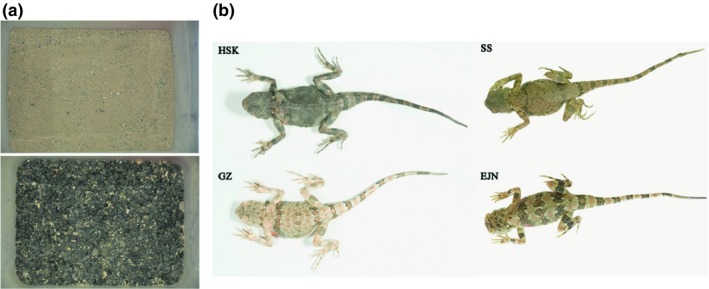
Feeding and reciprocal translocation experiments in artificial laboratory conditions. (a) Weathered yellow substrate (up) and black substrate (down) used in the reciprocal translocation experiments. (b) Photograph of lizards for melanic HSK and nonmelanic GZ populations after 60 days translocation and for EJN and SS populations after 30 days treatment

We used rectangular light plastic boxes (0.79 m × 0.60 m × 0.50 m) to house captured lizards. Each box was covered by 3‐ to 5‐cm‐thick sand and stones, that is, using black versus weathered yellow stones collected from natural habitats of populations to, respectively, simulate natural dark versus light habitat types (Figure 2a). Partial regions of each box were in direct sunlight for 1–2 hr during the day to allow lizards' body temperature thermoregulation. Lizards were fed daily with *Tenebrio molitor* larvae and had a permanent supply of water. Prior to experiments, all melanic and nonmelanic *P. versicolor* adults were regularly fed in black and weathered yellow habitat, respectively. During the translocation experiments, melanic population was housed in a weathered yellow sand habitat, while the nonmelanic populations were kept in black substrate. Each box housed eight to nine individuals. Males and females of the same population were allocated evenly in independent boxes. A total of 45 melanic *P. versicolor* adults (HSK population, 18 males, 27 females) and 78 nonmelanic adults (EJN: 11 males, 23 females; SS: 11 males, 33 females) from the first sampling trip were fed over one month. In addition, 24 melanic adults (HSK population, 6 males, 18 females) from the second sampling trip were raised over two months.

### 2.3 Dorsal color measurements

Comparisons of dorsal color between melanic and nonmelanic populations were conducted independently through two sets of measurements. The first set was to measure reflectance of HSK and EJN/SS populations using the spectrophotometer. The second was to analyze RGB, and luminance values estimated from photographs of other individuals of HSK and GZ populations using Adobe Photoshop SC6. RGB values provide color information which could show additional variation in hue compared with dark lizards. The former method using fiber spectrophotometer is very sensitive for color changes and easy to finish white color calibration. Moreover, the reflective probe should be very close to the measuring object in the presence of alternating current during measurement. It is thus good to be used in laboratory environment. However, it has disadvantages to obtain coloration data on the whole body conveniently. In contrast, the latter method has the obvious advantage to measure dorsal and substrate color immediately and conveniently during field work.

A spectrophotometer (AvaSpec‐2048), which comprises a mainframe (AvaSpec‐2048‐USB2), a photosource (AvaLight‐DH‐S), a reflective probe (FCR‐7UV400‐2‐ME), a probe fixator (RPH‐1), and a white diffusing panel (WS‐2) used for reflective calibration before measuring, was used to record the skin luminous reflectance (%) of 12 measuring points (Figure 1a), and these measuring were described by Tong et al. (2016). Measurements were performed under the same photosource without the influence of natural light. Sampling interval of spectrophotometer is 0.60 nm, and wavelength range is 200–1,100 nm (Yang, Cai, & Liang, 2011) which includes the visual range (300–700 nm) of Squamata and their potential avian predators (Bennett & Cuthill, 1994). The probe fixator was applied to fasten reflective probe. We then used the white diffusing panel, for calibration after preheated the mainframe. Measurements were taken with the probe held at a 90° angle and 2 mm above the lizard's skin. Exposure time was set to 100 ms.

A digital camera (D7100, Nikon) was fixed with tripod and held at 90° angle to the subject to take the photographs. A ColorChecker Digital SG (X‐rite, Michigan) calibration target was used to test the uniformity of the lighting environment. Detailed methods were described by Mckay (2013). Digital photographs for each of 24 HSK lizards (6 males, 18 females) and 20 GZ lizards (11 males, 9 females) were firstly taken under two 36‐W full‐spectrum fluorescent bulbs (5500K, 96 CRI) free from the effects of other illumination with photographic parameters (ISO 250, 1/80 shutter speed, 5.3 aperture value with white balance for direct sunlight) within 24 hr after captured. The distance of the front surface of the camera lens to shooting background surface was set to approximately 24.4 cm. Both bulbs were separated placed at two sides of the lizard body trunk but slight above the camera with the same height to the background and with approximate 6 cm horizontal distance to the camera in vertical direction to the lizard trunk. The photographs of the 24 melanic HSK lizards were taken again over two months after substrate translocation. Each lizard was photographed on a clean piece of A4 paper and finished within 5 s. Photographs were analyzed to quantify coloration based on the established methods (Villafuerte & Negro, 1998; Westland, Ripamonti, & Cheung, 2012).

To understand the body color variation in response to substrate color within a relatively short (24 hr) and long (30 days) time, the spectrophotometer was used in measuring lizards’ body color. Each lizard was immediately fed in a weathered yellow (for HSK population) or black (for SS and EJN populations) sand habitat after we finished its first measurement (set as 0 hr). Subsequently, two measurements were carried out in the 24th hour (as changing color within 24 hr can be negligible for this species based on our preliminary field observation in HSK and GZ populations, here we only measured ten lizards randomly selected from each group) and 30th day, respectively. The body color of 11 individuals (3 HSK, 3 EJN, 3 SS individuals in 30 days treatment, and 2 HSK in 2 months treatment) not in good body condition was only measured at 0 hr. All the measurements were performed from 9:00 a.m. to 11:00 a.m. with the room temperature of 25 ± 2°C.

### 2.4 Photographs of substrate and lizards in field

To compare the dorsal color variation between black and weathered yellow substrates, a total of 20 and 24 digital photographs from the HSK and the GZ areas, respectively, were taken. Since no significant color variation existed among different nonmelanic populations (GZ, SS, and EJN) or their substrates (Figures 1a and 2b), here we only used GZ population which was geographically closest to the HSK lizards. Each digital photograph represents the place of substrate where lizards were discovered. Another 10 digital photographs with each of them containing both a lizard and the substrate where it was captured were taken from each of HSK and GZ to analyze the relationship between dorsal color and substrate color. All these photographs were taken from 9:00 a.m. to 11:00 a.m. within one day of fieldwork in July 2018. A black umbrella was used to block the direct sunlight. All these photographs were taken by the same camera with identical photographic parameters and were analyzed with the same color calibration described above.

### 2.5 Substrate selection

**Figure 3:**
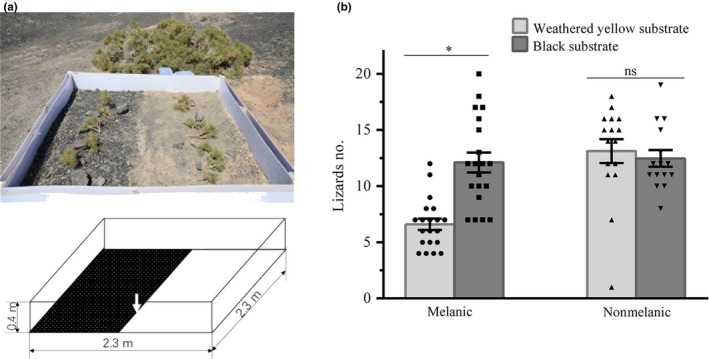
Substrate color selection. (a) Schematic diagram of the choice experiment used to test whether melanic and nonmelanic *Phrynocephalus versicolor* adults are behaviorally segregated according to their color. This area is divided into two type of substrates: where black surface indicates black substrate (left), and white surface represents weathered yellow substrate (right), and lizards were released at the boundary of two substrates (pointed by the arrow). See text for details. (b) Differences of lizards counting between black substrate and weathered yellow substrate for melanic or nonmelanic lizards. Statistics are presented as mean ± *SEM*. *Significant difference (*p* < .05) reported for corresponding color measuring point. ns, not significant

To investigate whether melanic HSK and nonmelanic GZ *P. versicolor* adults were behaviorally segregated based on substrate color preference, according to their dorsal coloration, a choice experiment was performed to allow lizards choose between dark or light substrate. We conducted experiments at sampling site in HSK, where only black substrates are found. Polystyrene cystosepiment (2.3 m in length and 0.4 m in height) was vertically placed to form a square area, and sand was stacked outside of this plastic board to reinforce it; half of this square area (5.29 m^2^) was covered with weathered yellow substrates collected from GZ to simulate natural light substrate types after moving 3–5 cm thick of original black sands and stones (Figure 3a). A total of 24 melanic (12 males, 12 females) or 26 (13 males, 13 females) nonmelanic lizards were released at the boundary of two types of substrates (pointed by the arrow in Figure 3a) one by one within one minute. After one hour of adaptation, we record the number of lizards in black and weathered yellow substrates every 15 min. After every three recording, we recaptured all the lizards and released at the substrate boundary by the same way. Two groups of lizards, using the same substrate area, were tested separately in two different days of fieldwork from 8:30 a.m. to 11:30 a.m. and from 3:30 p.m. to 7:30 p.m. Finally, a total of 20 and 15 times of recordings were, respectively, made for melanic and nonmelanic group, and were used for further analysis.

### 2.6 Data analysis

The spectroscopic data, which included reflectance values with wavelength ranging from 300 to 700 nm, were extracted and switched through the bundled software AvaSoft 8.1 and used for the following analysis. A single linear mixed model analysis where the reflectance of all body parts was entered as dependent variable and individual identity is used as random factor was performed (a) between melanic and nonmelanic lizards for pretreatment or post‐treatment of translocation (fixed effect was lizards color); (b) between pretreatment and post‐treatment of translocation for melanic or nonmelanic lizards (fixed effect was treatment time); and (c) between female and male lizards for melanic or nonmelanic before treatment (fixed effect was sex). For each point (M1–M12, see Figure 1a), we analyze the reflectance differences (a) between melanic and nonmelanic lizards for pretreatment or post‐treatment of translocation using independent sample *t* test and (b) between pretreatment and 24 hr/30 days of translocation treatment for melanic or nonmelanic lizards using paired *t* test.

For all the photographs, RGB (Red, Green, Blue) values and luminance of lizards/substrates were obtained from 1 cm^2^ of skin/16 cm^2^ substrate (including four 4 cm^2^ squares) using Adobe Photoshop SC6 following the methods described by Stevens et al. (2007). RGB and luminance values acquired from same lizard/substrate were averaged and used for subsequent statistical analysis. R, G, and B values were further illustrated by three‐dimensional scatterplots. We further compared (a) the differentiation of RGB and L values between black and weathered yellow substrate, and that between melanic and nonmelanic lizards, using independent sample *t* test and (b) the change in lizard number between black and weathered yellow substrates in substrate preference experiments using paired *t* test. All data met the assumption of normality. Prior to linear mixed model analysis and *t* tests, data were further tested for variance homogeneity using Levene's tests. To meet these requirements, reflectance data were square‐root transformed. Correlations between all lizards’ dorsal coloration and all substrate color were conducted by Pearson's correlation coefficient. Statistics were generally presented as mean ± *SEM*, with significance reported when *p* < .05. All analyses were performed using the statistical software system SPSS v.20.

## 3 RESULTS

### 3.1 Color variation of substrates and lizards

Dorsal and substrate color were compared between the geographically nearby melanic HSK population and the nonmelanic GZ population. The luminance value for black substrate (HSK, 110.53 ± 1.82)/melanic lizards (HSK population, 110.90 ± 15.08) showed significantly lower than that of weathered yellow substrates (GZ, 127.01 ± 0.82)/nonmelanic lizards (GZ population, 155.91 ± 10.85), respectively, based on independent sample *t* test (substrates: *t*_42_ = 8.720, *p* < .001; lizards: *t*_42_ = 41.201, *p* < .001). Moreover, significant differentiation on RGB values between two types of substrates (Red: *t*_42_ = 8.670, *p* < .001; Green: *t*_42_ = 8.727, *p* < .001; Blue: *t*_42_ = 7.134, *p* < .001) and that between melanic and nonmelanic populations (Red: *t*_42_ = 10.449, *p* < .001; Green: *t*_42_ = 10.702, *p* < .001; Blue: *t*_42_ = 5.364, *p* < .001) was obtained by independent sample *t* test. The RGB values between groups of substrates/lizards were further illustrated by separated clusters in the three‐dimensional scatter plot (Appendix S1).

Significantly positive correlation of color variation between all lizards and all substrates was detected based on Pearson's correlation coefficient analysis (luminance: *r* = .761, *p* < .001, *n* = 20; Red: *r* = .640, *p* = .002, *n* = 20; Green: *r* = .587, *p* = .006, *n* = 20; Blue: *r* = .673, *p* = .001, *n* = 20).

### 3.2 Reflectance variation between melanic and nonmelanic populations

Before the treatment of substrate translocation, no significant differences of reflectance between male and female (melanic: *F*_1, 538_ = 2.044; *p* = .153; nonmelanic: *F*_1, 934_ = 0.012; *p* = .912) were detected, and we therefore did not perform separately statistical analysis for each gender in the following study. The reflectance of nonmelanic lizards (EJN and SS populations) was significantly higher than that of melanic lizards (HSK population; *F*_1, 1452_ = 310.734, *p* < .001).

After 30 days of weathered yellow substrate treatment for HSK lizards, their reflectance was still significantly lower than that of untreated EJN and SS lizards (*F*_1, 1416_ = 307.528, *p* < .001). For EJN and SS lizards, after 30 days of black substrate treatment, the reflectance was significantly higher than that of untreated HSK lizards (*F*_1, 1380_ = 473.052, *p* < .001).

### 3.3 Effects of habitat color on body color variation

For HSK population, no significant reflectance differences were found by single linear mixed model analysis between 0 hr and 24th hour (*F*_1, 238_ = 0.283; *p* = .595) or that between 0 hr and 30th day of weathered yellow substrate treatment (*F*_1, 1020_ = 0.240; *p* = .624), though two measuring points (M1 and M6) experienced slight but significant color change after 30 days of weathered yellow substrate treatment (Appendix S2).

For nonmelanic populations (SS and EJN), the reflectance values did not show significant differentiation after 24 hr (*F*_1, 238_ = 0.149; *p* = .700) or 30 days (*F*_1, 1776_ = 2.372; *p* = .124) of substrate translocation treatment when compared with that of pretreatment. Among them, four measuring points (M3, M4, M9, and M10) showed small but significant statistic differences based on paired *t* test analysis (Appendix S2). Neither group of lizards showed macroscopic variation on their body coloration after translocation treatment of substrates.

After two months of light substrate treatment, the original luminance value (110.90 ± 15.08) of 24 HSK melanic adults significantly increased to 130.60 ± 11.35 (*F*_2, 44_ = 24.701, *p* < .001), and the corresponding RGB values were also increased (Red: *F*_2, 44_ = 23.425, *p* < .001; Green: *F*_2, 44_ = 49.089, *p* < .001; Blue: *F*_2, 44_ = 93.323, *p* < .001). However, the luminance value of 24 HSK adults after two months treatment was still significant smaller than the initial luminance value (155.91 07 ± 18.547) of the nearby GZ population (*F*_2, 40_ = 54.324, *p* < .001).

### 3.4 Substrate color selection

Melanic lizards tended to prefer the black rather than weathered yellow substrate (*t*_19_ = 6.200; *p* < .001), while no significant preference of substrate color was detected for nonmelanic GZ lizards (*t*_14_ = 1.025; *p* = .323; Figure 3b), based on paired *t* tests.

## 4 DISCUSSION

Using spectrometry technology and digital photography, we analyzed for the first time the detailed body coloration and behavior responses of different *P. versicolor* populations to substrate color. We found that the body coloration of *P. versicolor* from dark substrate was conspicuously melanic compared with nonmelanic conspecifics from light substrate, whether they experienced the substrate translocation treatment or not. The lizards had little changes on the reflectance of their dorsal skin, and no macroscopic color changes were observed after translocation treatment of substrates. Also, melanic lizards prefer to live in black substrate, but nonmelanic lizards are free of bias for substrate color.

Variation of animal coloration has been described to be correlated with climatic factors, such as the description of Gloger rule, which states that more heavily pigmented forms within one species tend to be found in more humid environment. However, it is often considered to be associated with substrate color, that is, the lizard color variation in white sands (Rosenblum et al., 2010). Unlike the EJN, SS, and GZ populations of *P. versicolor* with light dorsal coloration that inhabit the Gobi area with weathered yellow substrate, melanic *P. versicolor* living at HSK areas possess distinctly darker body color, revealed by our reflectance values, luminance values, and RGB values. And the extremely striking difference in substrate color between HSK and the other three areas (GZ, SS, and EJN; Figure 1) would most likely be the primary factor, though temperature (Krohn & Rosenblum, 2016; Rosenblum, 2005) and stress (Krohn & Rosenblum, 2016) may affect lizards' body color. Nonmelanic populations from large areas with variable elevation‐associated environmental conditions (Figure 1b) show similar light dorsal color, while the melanic population in HSK exhibits dark dorsal color in high altitude as well (Figure 1a), indicating that elevation is probably not contributing to explain the variance of coloration. Moreover, substrate color difference has been previously found to explain coloration variation within a species between geographically nearby populations, for example, *Anolis carolinensis* (Macedonia, Echternacht, & Walguarnery, 2003), *Crotalus lepidus lepidus* (Farallo & Forstner, 2012), and *Montivipera raddei* species complex (Rajabizadeh et al., 2015). We propose that this is the same case for melanic HSK and nonmelanic GZ populations.

Body coloration in many poikilothermic animals (with variable internal body temperature) is generally plastic, which may show macroscopic changes within a few hours or even a few seconds, such as *Paracheirodon innesi*, (Nagaishi, Oshima, & Fujii, 1990), *Pentapodus paradiseus* (Mäthger, Land, Siebeck, & Marshall, 2003), *Hyla japonica* (Choi & Jang, 2014), and *Hoplolatilus chlupatyi* (Goda, 2017). Surprisingly, the melanic *P. versicolor* did not increase their reflectance value after 24 hr of treatment in weathered yellow substrate, and the same results were obtained for nonmelanic populations which did not decrease their reflectance value after translocation to dark substrate. Moreover, the melanic and nonmelanic populations still, respectively, kept dark and light dorsal color after 60 or 30 days of translocation treatment of substrates (Figure 2), showing slight phenotypic plasticity on dorsal color could occur in this case. This finding support that dorsal color variation in *P. versicolor* is inclined to morphological adaptation rather than physiological plasticity. However, it is worthy to note that several measuring points showed significantly statistical differences between pretreatment and post‐treatment, and we therefore speculate that it may be induced by a certain degree of aggregation and dispersion of melanin granules in these body parts. As this work is primarily focused on ecological expectations of body coloration in *P. versicolor*, this scenario could be explored in the future with tissue experiments, where the number of chromatophores in corresponding parts of tissues is measured.

The melanic degree of animal skin is often associated with the activity of melanophores (Alibardi, 2013), and it may take several days or even month for epidermis melanophore layer to divert melanosome to adjacent keratinocyte layer, rendering the skin melanism, that is, morphological color change (Cooper & Greenberg, 1992). Selective stress may influence the regulation of hormones and other potential genetic factors that are important for pigment synthesis for example, α‐MSH (Fernandez & Bagnara, 1991; Mashinini, Heideman, & Mouton, 2008). In several classic studies, individual body coloration would become similar with local substrate environment when they subject to stronger pressure from predators (Cooper & Allen, 1994; Johnsson & Kjällman‐Eriksson, 2008; Kettlewell, 1973). Therefore, this could be the case for HSK *P. versicolor* populations since no melanic individuals were found outside of this area, whose dark body color tends to be influenced by evolutionary factors caused by long‐term substrate color stimuli, rather than the physiological changes in response to short‐term substrate color changes. Further research is needed to investigate the predator pressure and the underlying genetic factors that might induce the geographical dorsal color variation of *P. versicolor* populations.

In animals, behaviorally mediated background matching is a critical aspect for their successful survival in a new place, as they tend to choose a substrate that matches their body coloration (Banos‐Villalba, Quevedo, & Edelaar, 2018; Johansson & Nilsson‐Ortman, 2013; Kang et al., 2015). It exists in melanic *P. versicolor* lizards, as revealed by the significantly larger number of melanic individuals chose black substrate than weathered yellow substrate (Figure 3), suggesting a segregated behavior according to their melanic body coloration existed in this population. In *Phrynocephalus* lizards, the melanic part body coloration may contribute to the thermal regulation for viviparous species that survive in cold environments of the Qinghai‐Tibet Plateau (QTP, Jin et al., 2016), for example, the functions of abdominal black‐speckled area reported in *P. theobaldi* (Jin & Liao, 2015). However, almost all melanic and nonmelanic *P. versicolor* populations only survive at dark and light substrates, respectively, and moreover, nonmelanic *P. versicolor* populations did not display obvious color differences in large different elevation/temperature environments. Therefore, the primary pressure for melanic lizards is black substrate rather than environmental temperature. Such type of body coloration, combined with their native substrate color and their behavior for background matching, could improve the effectiveness of successful camouflage. Notably, nonmelanic *P. versicolor* lizards did not seem to have preference for weathered yellow substrate, as the lizard frequency in both types of substrates is similar (Figure 3). These results may indicate that the behaviorally mediated substrate preference of melanic *P. versicolor* population may evolve from phenotypic plasticity on dorsal coloration in their initial colonization, similar with the dorsal plasticity of a population of side‐blotched lizard, *Uta stansburiana* (Corl et al., 2018).

In conclusion, we discovered that substrate color associated dorsal coloration of *P. versicolor* is most likely a morphological long‐term adaptation in response to heterogeneous substrate color, which could help their camouflage effectiveness. Our study firstly addressed the natural dorsal color variation among intraspecific populations of *Phrynocephalus*. Our findings provide insights into our understanding on why pigmentation of *Phrynocephalus* in dark substrate emerged and may be useful for further identification of potential genetic factors responsible for morphological adaptation in a genomic and functional way.

## CONFLICT OF INTEREST

None declared.

## AUTHOR CONTRIBUTIONS

HJT, JSL, YBW, GS, and YTJ contributed experiments, analyses, and materials; HJT, AGD, and YTJ wrote and revised the manuscript; YTJ designed the study; and WZ contributed materials.

## Data Availability

Data on geographical sampling locations and all measured dorsal and substrate color are deposited in FigShare (https://figshare.com/s/80ff7eb34767f2dda8ac). The corresponding authors are responsible for any personal requirement for the materials.
