# Supplementary material for: Effects of substrate color on intraspecific body color variation in the toad‐headed lizard, Phrynocephalus versicolor
Source: Ecol Evol. 2019 Aug 15;9(18):10253–62. doi: 10.1002/ece3.5545 (PMC6787858; doi:10.1002/ece3.5545)
Supplement: Supplementary file 1 [file ECE3-9-10253-s001.doc]

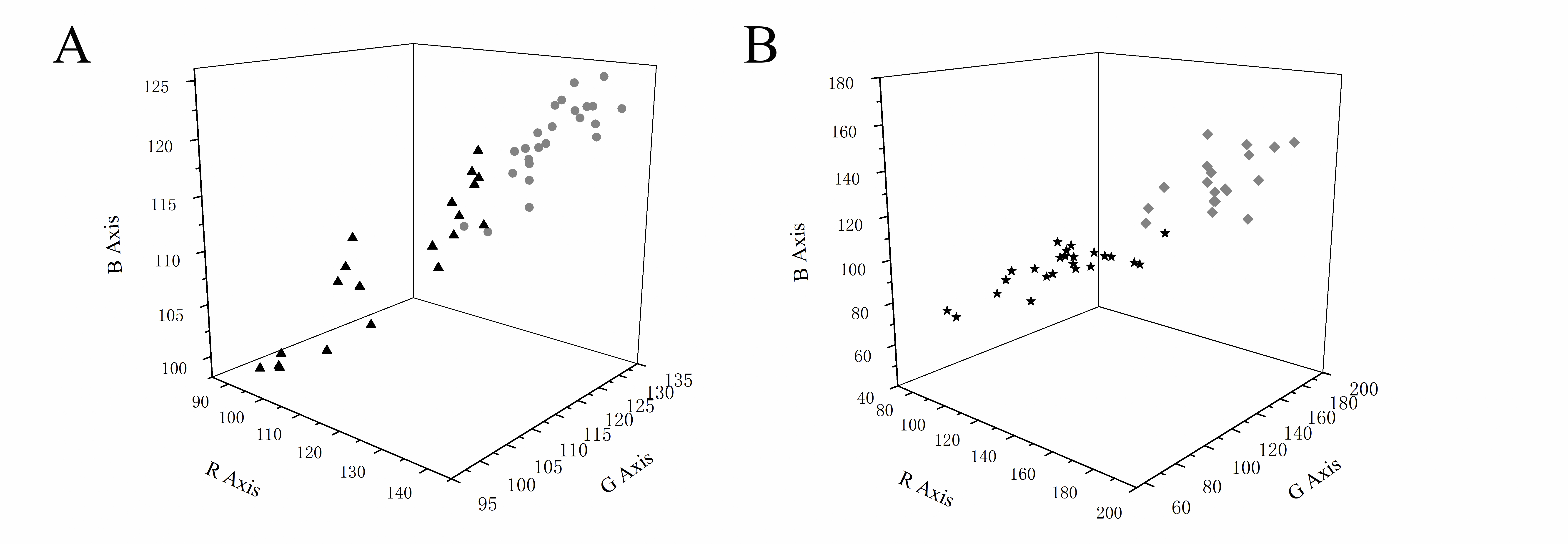


**Appendix S1.** Scatter plot of Red, Green and Blue values. A: RGB values measured from black (black triangles) and weathered yellow (gray circles) substrate. B: RGB values measured from the dorsal of melanic (black stars) and non-melanic (gray squares) *P. versicolor* adults.
