# Supplementary material for: Effects of substrate color on intraspecific body color variation in the toad‐headed lizard, Phrynocephalus versicolor
Source: Ecol Evol. 2019 Aug 15;9(18):10253–62. doi: 10.1002/ece3.5545 (PMC6787858; doi:10.1002/ece3.5545)
Supplement: Supplementary file 2 [file ECE3-9-10253-s002.doc]

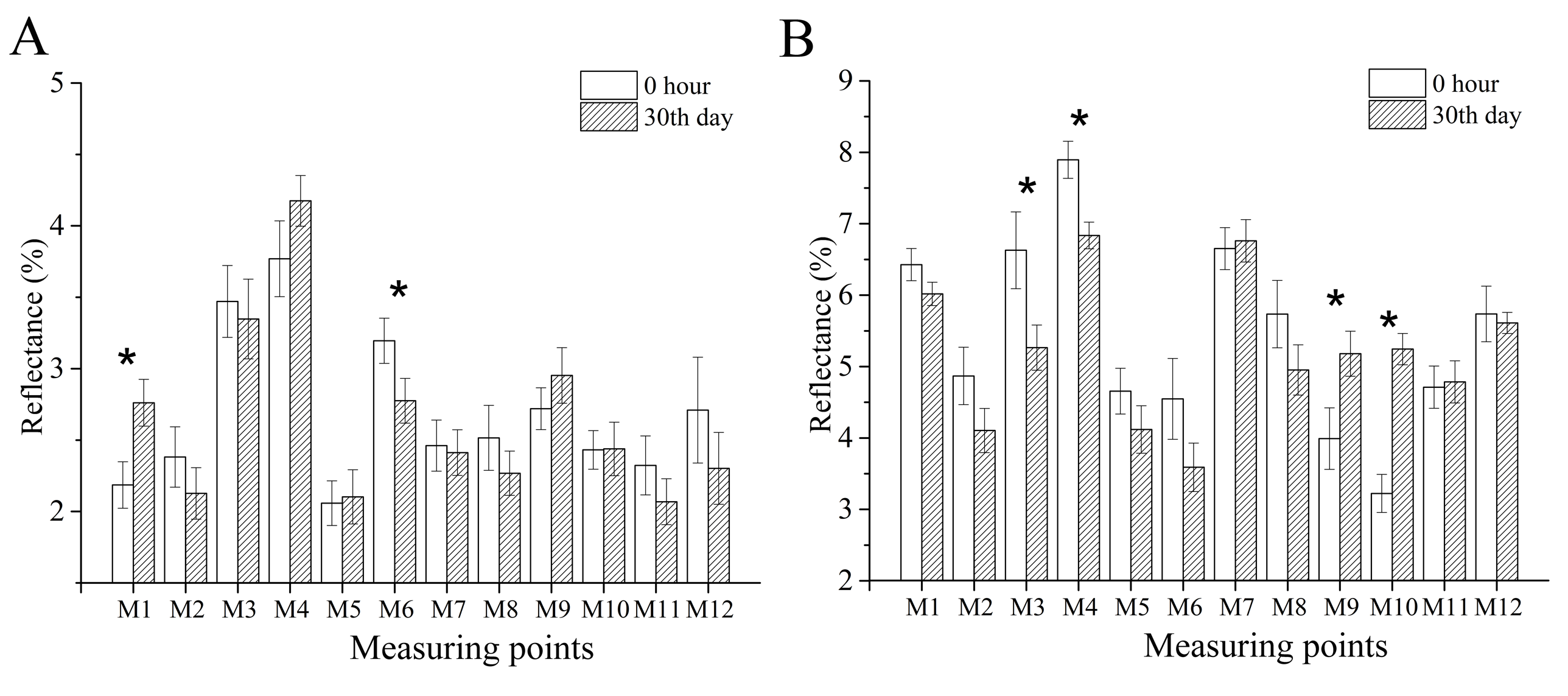


**Appendix S2.** Reflectance values (%) of each measuring point (M1-M12) for lizards. A: Reflectance (%) differences between 0 hour and 30 days treatment of weathered yellow substrates for HSK population. B: Reflectance (%) differences between 0 hour and 30 days treatment of black substrate for EJN and SS populations. Statistics are generally presented as mean ± SE. Asterisks represent significant differences (*P* < 0.05) reported for corresponding measuring points.
